# WITHDRAWN: The effect of ischemia on expression quantitative trait loci (eQTL) in human myocardium and insights into myocardial injury etiology

**DOI:** 10.21203/rs.3.rs-3967889/v2

**Published:** 2024-02-26

**Authors:** Azam Yazdani, Sameeksha Tiwari, Mahyar Heydarpour

## Abstract

26 February, 2024. Research Square has withdrawn this preprint as it was submitted and made public without the full consent of all the authors and without the full consent of the principle investigator of the registered clinical trial. Therefore, this work should not be cited as a reference.

